# Supplementary material for: Development of a Novel Live Attenuated Influenza A Virus Vaccine Encoding the IgA-Inducing Protein
Source: Vaccines (Basel). 2021 Jun 27;9(7):703. doi: 10.3390/vaccines9070703 (PMC8310050; doi:10.3390/vaccines9070703)
Supplement: Supplementary file 1 [file vaccines-09-00703-s001.zip › vaccines-1259195-supplementary.pdf]

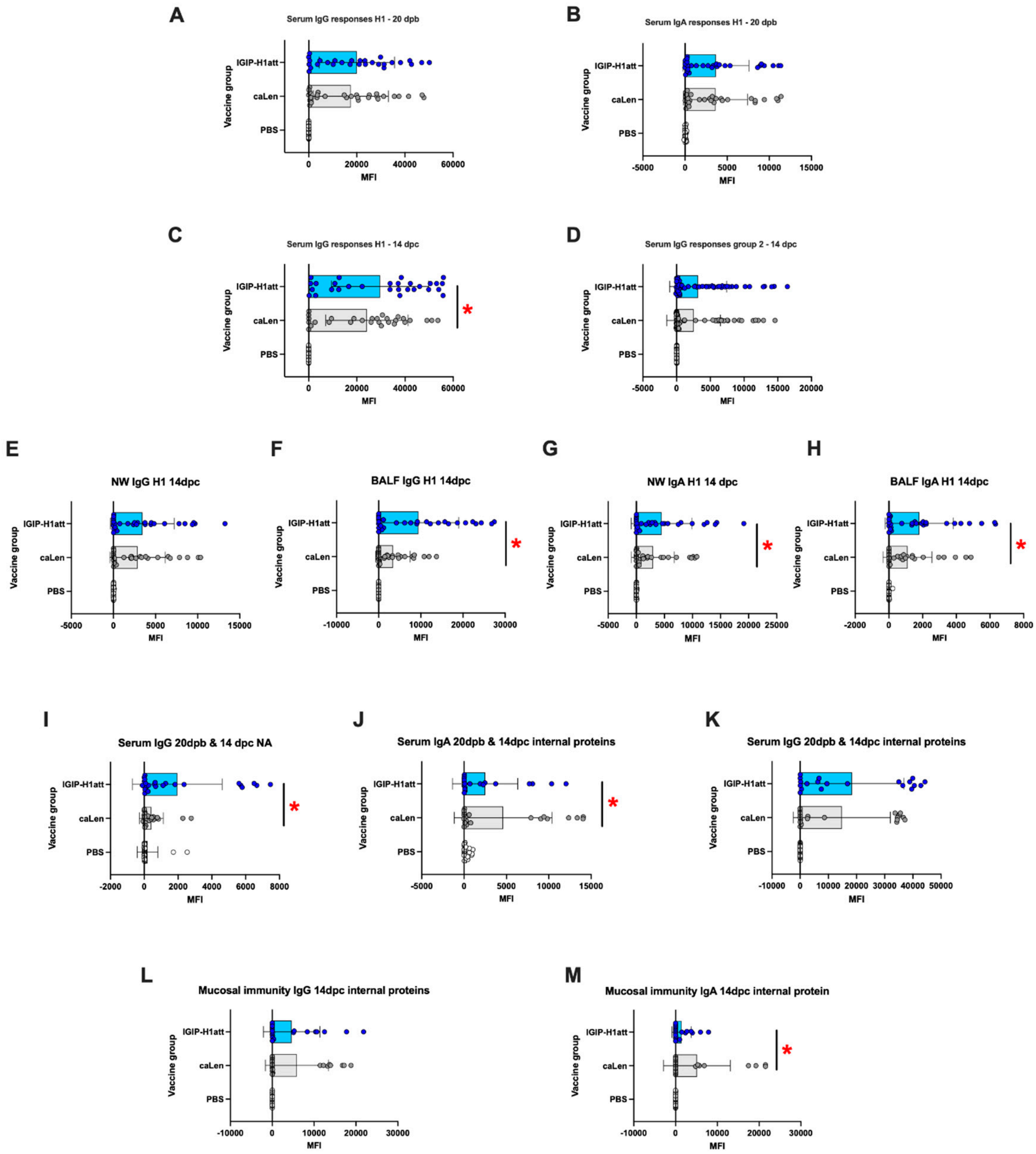

Figure S1. Influenza antigen microarray data combined. The combined data including the different antigens was plotted. Serum (**A**) IgG and (**B**) IgA at 20dpb against H1. Serum (**C**) IgG and (**D**) IgA at 14dpc against H1. (**E–F**) IgG in (**E**) NW and **(F**) BALF against H1 at 14dpc. (**G–H**) IgA in (**G**) NW and (**H**) BALF against H1 at 14dpc. (**I**) Serum IgG against NA. (**J–K**) Serum (**J**) IgA and (**K**) IgG against internal proteins. (**L–M**) Mucosal (**L**) IgG and (**M**) IgA against internal IAV proteins. Statistically significant differences between IGIP-H1att and caLen marked with red asterisks.

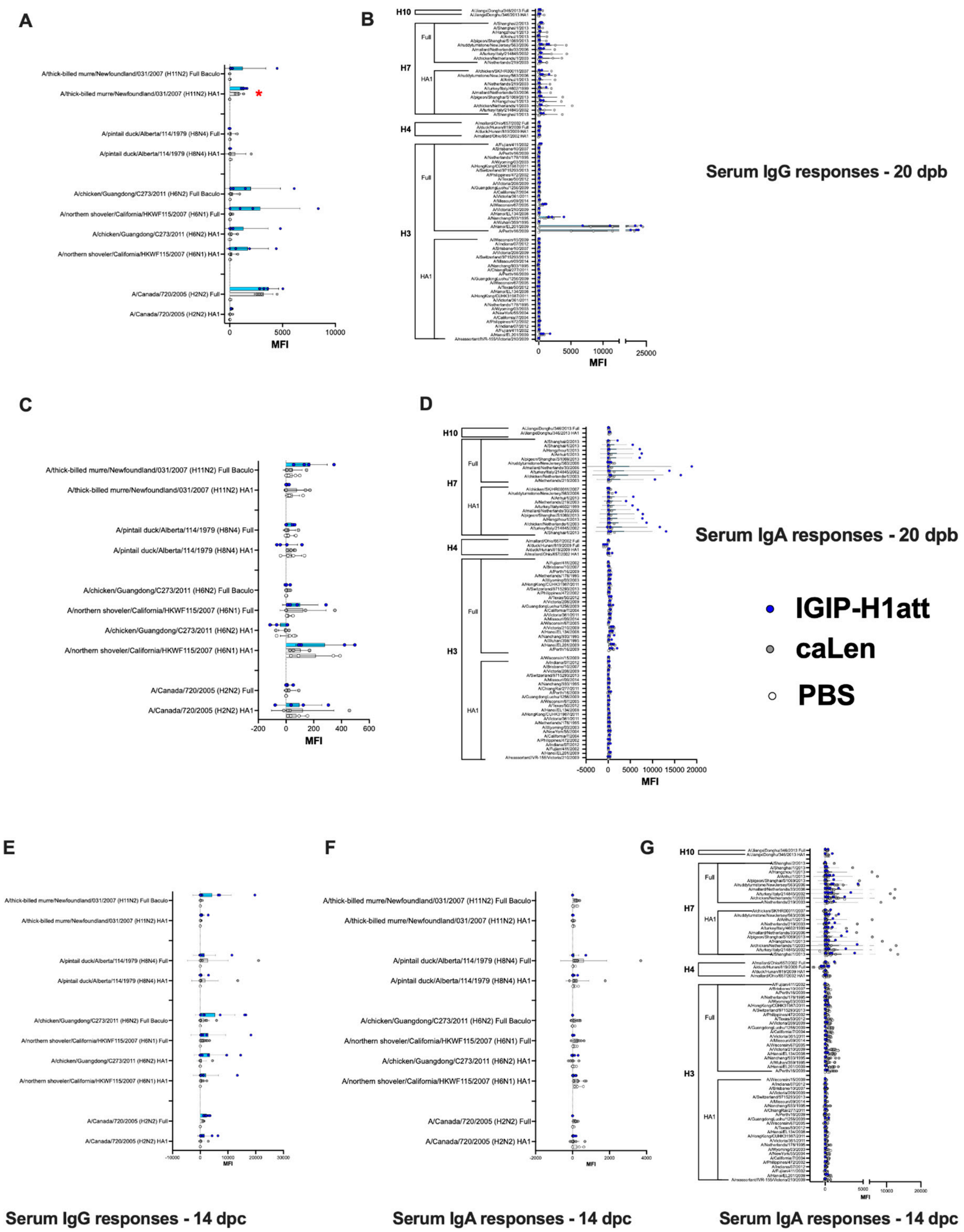

Figure S2. Influenza antigen microarray data against group 1 HA (H2, H6, H8 and H11) and group 2 HA (H3, H4, H7 and H10). (**A–B**) IgG against (**A**) group 1 and (**B**) group 2 HAs at 20dpb. (**C–D**) IgA against (**C**) group 1 and (**D**) group 2 HAs at 20dpb. (**E**) Serum IgG responses against group 1 at 14dpc. (**F–G**) Serum IgA response against (**F**) group 1 and (**G**) group 2 HAs. Statistically significant differences between IGIP-H1att and caLen are depicted red asterisks.

Table S1. Origin and catalog number of the antigens used in the Influenza antigen microarray†.

Isolate	Type of antigen	Catalog number
A/California/04/2009 (H1N1)	HA	11055-V08B
A/NewYork/18/2009 (H1N1)	HA	40009-V08H
A/England/195/2009 (H1N1)	HA	40005-V08H
A/California/07/2009 (H1N1)	HA	11085-V08H
A/Texas/05/2009 (H1N1)	HA	40006-V08H
A/Ohio/07/2009 (H1N1)	HA	40007-V08H
A/California/06/2009 (H1N1)	HA	40350-V08B
A/Beijing/22808/2009 (H1N1)	HA	40035-V08H
A/Brisbane/59/2007 (H1N1)	HA	11052-V08H
A/SolomonIslands/3/2006 (H1N1)	HA	11708-V08H
A/Ohio/UR060991/2007 (H1N1)	HA	11687-V08H
A/PuertoRico/8/1934 (H1N1)	HA	11684-V08H
A/Canada/720/2005 (H2N2)	HA	11688-V08H
A/HongKong/483/1997 (H5N1)	HA	11689-V08H
A/goose/Guiyang/337/2006 (H5N1)	HA	11690-V08H
A/japanesewhiteeye/HongKong/1038/2006 (H5N1)	HA	11694-V08H
A/chicken/India/NIV33487/2006 (H5N1)	HA	11712-V08H
A/Cambodia/R0405050/2007 (H5N1)	HA	11710-V08H
A/Vietnam/1194/2004 (H5N1)	HA	11062-V08H1
A/duck/Hunan/795/2002 (H5N1)	HA	11698-V08H
A/whooperswan/Mongolia/244/2005 (H5N1)	HA	11709-V08H
A/Egypt/2321NAMRU3/2007 (H5N1)	HA	11697-V08H
A/northernshoveler/California/HKWF115/2007 (H6N1)	HA	11723-V08H
A/chicken/Guangdong/C273/2011 (H6N2)	HA	40398-V08B
A/pintailduck/Alberta/115/1979 (H8N4)	HA	11722-V08H
A/shorebird/Delaware/261/2003 (H9N5)	HA	40181-V08B
A/HongKong/1073/1999 (H9N2)	HA	11229-V08H
A/chicken/HongKong/G9/1997 (H9N2)	HA	40036-V08H
A/guineafowl/HongKong/WF10/1999 (H9N2)	HA	11719-V08H
A/HongKong/3239/2008 (H9N2)	HA	40178-V08B
A/HongKong/35820/2009 (H9N2)	HA	40174-V08B
A/thickbilledmurre/Newfoundland/031/2007 (H11N2)	HA	40187-V08B
A/Perth/16/2009 (H3N2)	HA	40043-V08H
A/Hanoi/EL201/2009 (H3N2)	HA	40490-V08B
A/Wuhan/359/1995 (H3N2)	HA	40475-V08B
A/Nanchang/933/1995 (H3N2)	HA	40485-V08B
A/Hanoi/EL134/2008 (H3N2)	HA	40489-V08B
A/Victoria/210/2009 (H3N2)	HA	40058-V08B
A/Wisconsin/67/2005 (H3N2)	HA	11972-V08H
A/Missouri/09/2014 (H3N2)	HA	40494-V08B
A/Victoria/361/2011 (H3N2)	HA	40145-V08B
A/California/7/2004 (H3N2)	HA	40118-V08B
A/GuangdongLuohu/1256/2009 (H3N2)	HA	40152-V08B
A/Victoria/208/2009 (H3N2)	HA	40151-V08B
A/Texas/50/2012 (H3N2)	HA	40354-V08B
A/Philippines/472/2002 (H3N2)	HA	40487-V08B
A/Switzerland/9715293/2013 (H3N2)	HA	40497-V08B

A/HongKong/CUHK31987/2011 (H3N2)	HA	40146-V08B
A/Wyoming/03/2003 (H3N2)	HA	11715-V08H
A/Netherlands/178/1995 (H3N2)	HA	40486-V08B
A/Brisbane/10/2007 (H3N2)	HA	11056-V08H
A/Fujian/411/2002 (H3N2)	HA	40488-V08B
A/duck/Hunan/819/2009 (H4N2)	HA	40390-V08B
A/mallard/Ohio/657/2002 (H4N6)	HA	11714-V08H
A/Netherlands/219/2003 (H7N7)	HA	11082-V08B
A/chicken/Netherlands/1/2003 (H7N7)	HA	11212-V08B
A/turkey/Italy/214845/2002 (H7N3)	HA	40128-V08B
A/mallard/Netherlands/33/2006 (H7N8)	HA	40172-V08B
A/ruddyturnstone/NewJersey/563/2006 (H7N2)	HA	40170-V08B
A/pigeon/Shanghai/S1069/2013 (H7N9)	HA	40106-V08H
A/Anhui/1/2013 (H7N9)	HA	40103-V08H
A/Hangzhou/1/2013 (H7N9)	HA	40105-V08H
A/Shanghai/2/2013 (H7N9)	HA	40239-V08H
A/JiangxiDonghu/346/2013 (H10N8)	HA	40359-V08B
A/Egypt/2321NAMRU3/2007 (H5N1)	NA	40045-VNAHC
A/HongKong/4801/2014 (H3N2)	NA	40569-V07H
A/Aichi/2/1968 (H3N2)	NA	40199-V07H
A/California/04/2009 (H1N1)	NA	11058-V08B
A/Hubei/1/2011 (H5N1)	NA	40018-V07H
A/Anhui/1/2013 (H7N9)	NA	40108-V07H
A/PuertoRico/8/1934 (H1N1)	NA	40196-VNAHC
A/Michigan/45/2015 (H1N1)	NA	40568-V07H
A/Anhui/1/2005 (H5N1)	NA	11676-VNAHC
A/Babol/36/2005 (H3N2)	NA	40017-V07H
A/USSR/90/1977 (H1N1)	NA	40197-V07H
A/California/07/2009 (H1N1)	NP	40205-V08B
A/Puerto Rico/8/1934 (H1N1)	NP	11675-V08B
A/Hong Kong/1/1968 (H3N2)	NP	40208-V08B
A/Aichi/2/1968 (H3N2)	NP	40207-V08B
A/Aichi/2/1968 (H3N2)	M1	40215-V07E
A/Puerto Rico/8/1934 (H1N1)	M1	40010-V07E
A/Brevig Mission/1/1918	M1	40211-V07E
A/Puerto Rico/8/1934 (H1N1)	NS1	40011-V07E
A/Puerto Rico/8/1934 (H1N1)	NS2	40012-VNA

†All antigens obtaiend from Sino Biological (Wayne, PA).
